# Ultra Short Osteotomy With Mortise‐Tenon Diaphyseal Prosthesis Reconstruction for Forearm Metastases: A Retrospective Study

**DOI:** 10.1111/os.70321

**Published:** 2026-04-20

**Authors:** Yunxiu Chen, Tongfu Wang, Miaomiao Gao, Xiaoyi Wang, Chengke Li, Jingyu Zhang

**Affiliations:** ^1^ Department of Orthopedic Surgery The Affiliated Hospital of North China University of Science and Technology Tangshan Hebei China; ^2^ Tianjin Hospital, Tianjin University Tianjin China

**Keywords:** diaphyseal prosthesis, forearm, metastatic lesions, mortise‐tenon, radius, ulna

## Abstract

**Objective:**

Forearm diaphyseal metastases are rarely encountered and are predisposed to pathological fracture because torsional stress is repeatedly generated during pronation–supination. In addition, relatively long osteotomy is often required by conventional intercalary prostheses, and limited bone stock in the radius and ulna may be sacrificed. In this study, the feasibility and short‐ to mid‐term outcomes of an intercalary mortise–tenon diaphyseal prosthesis designed to enable ultra‐short osteotomy were evaluated in patients undergoing reconstruction after en bloc resection of forearm metastases.

**Methods:**

Five consecutive patients with diaphyseal metastases of the forearm (radius, *n* = 3; ulna, *n* = 2) who were treated with en bloc resection and implantation of a custom mortise–tenon diaphyseal prosthesis between June 2019 and November 2023 were retrospectively reviewed. Pain and limb function were assessed using the visual analogue scale (VAS) and Musculoskeletal Tumor Society (MSTS) score, respectively. Osteotomy length, perioperative findings, complications, local recurrence, implant‐related events, and survival were recorded. Pre‐ and postoperative VAS and MSTS scores were compared using the Wilcoxon signed‐rank test.

**Results:**

A mean follow‐up of 40.2 months (range, 19–72) was achieved. A mean osteotomy length of 3.6 cm (range, 3.0–4.0) was recorded, and reconstruction was completed with limited bone resection. Pathological fractures were observed in three patients, whereas Mirel's scores of 10 were documented in the remaining two patients. At final follow‐up, the mean VAS score was reduced to 0.4 (range, 0–1), and the mean MSTS score was increased to 26.2 (range, 25–27); statistical significance was reached for both comparisons. The 12‐ and 24‐month survival rates were estimated at 100% and 80%, respectively. No local recurrence, implant failure, or major postoperative complications were observed during follow‐up.

**Conclusion:**

In this small retrospective case series, intercalary reconstruction using a mortise–tenon diaphyseal prosthesis after en bloc resection was shown to be feasible for forearm diaphyseal metastases, and substantial pain relief together with satisfactory functional recovery was achieved. Long‐term durability and oncologic outcomes should be confirmed in larger studies.

## Graphical abstract

A novel mortise‐tenon diaphyseal prosthesis enables ultra‐short osteotomy for forearm bone metastases, providing stable fixation with significant pain relief and functional recovery. This approach preserves bone stock and demonstrates excellent safety, with no implant failure, local recurrence, or major complications during mid‐ to long‐term follow‐up.

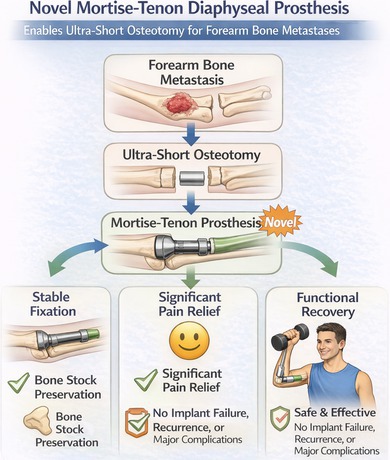

## 1 Introduction

Bone metastases are frequently encountered in malignant tumors, and an approximately 35–40‐fold higher incidence has been reported compared with primary malignant bone tumors [1]. Metastatic lesions typically occur in bones with abundant red marrow and trabecular structure, such as the spine, femur, and humerus [2, 3, 4]. In contrast, metastases of the forearm are exceedingly rare, accounting for only 0.01%–2% in the ulna and 0.4%–1% in the radius [5, 6, 7, 8, 9]. When metastases involve the radius or ulna, repetitive torsional loading during pronation–supination has been considered to predispose the affected segment to pathological fracture, by which subsequent management may be complicated and prognosis may be adversely affected [1, 10]. Accordingly, timely surgical management has been increasingly considered for metastatic lesions in the radius and ulna.

In this context, several surgical strategies have been utilized for forearm metastases, including intramedullary nail or plate fixation, lesion curettage with bone cement filling, en bloc resection with reconstruction, and, in selected cases, amputation [11, 12]. When fixation or intralesional procedures are performed, the tumor is inevitably violated, by which incomplete removal may be introduced and subsequent local recurrence may be facilitated. By contrast, improved local control has been reported with en bloc resection, and reconstruction of the resulting segmental defect is generally required to restore forearm function.

Common reconstruction options include vascularized fibula grafts, allografts, and endoprosthetic replacement [12, 13], and distinct advantages and limitations have been recognized for each approach [14, 15]. Although prosthetic reconstruction enables early limb mobilization and favorable outcomes [12, 16], evidence regarding prosthetic implantation for radial and ulnar diaphyseal metastases remains limited, largely because these lesions are rarely encountered. Unlike long bones, the radius and ulna are short, and metastatic lesions are generally small. With conventional diaphyseal prostheses, long connecting stems are typically required for fixation; consequently, extensive bone stock may need to be sacrificed and ultra‐short osteotomy may be difficult to achieve. To address this limitation, a diaphyseal prosthesis was developed with reference to the traditional mortise–tenon structure. By this design, stable fixation is intended to be achieved with ultra‐short segmental resection (approximately 3–4 cm), remaining bone stock can be preserved, and intraoperative assembly may be simplified because overlapping segments or intermediate screws are not required.

Accordingly, the present study was designed to describe the clinical application of en bloc resection followed by mortise–tenon diaphyseal prosthetic reconstruction for forearm diaphyseal metastases and to evaluate associated outcomes. A single‐center experience was summarized, and relevant literature was reviewed to contextualize this reconstructive strategy for these rarely encountered but clinically challenging lesions.

## 2 Materials and Methods

### 2.1 Inclusion and Exclusion Criteria

Inclusion criteria were as follows: (1) metastatic lesions involving the radial or ulnar diaphysis, (2) treatment with en bloc resection followed by implantation of a mortise–tenon diaphyseal prosthesis, (3) follow‐up evaluations available for outcome assessment.

Exclusion criteria were as follows: (1) poor general condition precluding tolerance of anesthesia or surgery, (2) pathological involvement of the ulnar or radial head.

### 2.2 Patient Characteristics

Between June 2019 and November 2023, five patients with forearm diaphyseal bone metastases (radius, *n* = 3; ulna, *n* = 2) were treated with en bloc resection followed by implantation of a mortise‐tenon diaphyseal prosthesis at the Department of Bone and Soft Tissue Oncology, a tertiary care hospital, China (Table 1). Three presented with pathological fractures. Pathological fractures were present in three patients. Upper‐limb pain and limitations in daily activities were reported by all patients at presentation. In all cases, metastatic lesions were located in the mid‐diaphyseal segment of the radius or ulna. The primary tumor origins included lung cancer (*n* = 2), breast cancer (*n* = 1), kidney cancer (*n* = 1), and multiple myeloma (*n* = 1).

**TABLE 1:** Clinical characteristics of patients.

Patient	Sex	Age (year)	Primary tumor	Location	Side	Pathological fracture	Mirel's score	Osteotomy length (cm)
1	F	65	Lung cancer	Radius	Left	Yes	—	3.0
2	F	61	Breast cancer	Ulna	Right	No	10	4.0
3	M	72	Lung cancer	Ulna	Right	Yes	—	4.0
4	F	69	Kidney cancer	Radius	Left	No	10	3.0
5	M	75	Multiple myeloma	Radius	Left	Yes	—	4.0

Abbreviations: F, female; M, male.

All procedures were performed by the same senior surgeon, ensuring consistency in surgical technique, while postoperative monitoring and follow‐up evaluations were carried out by the same researcher. This retrospective study was approved by the Ethics Committee of A tertiary care hospital in China (Ethics Approval Number: 2021 Medical Ethics Review 160), and written informed consent was obtained from all patients prior to inclusion.

### 2.3 Preoperative Examination

All patients were evaluated preoperatively to assess general condition and tolerance for anesthesia and surgery. Standard imaging workup included X‐ray and magnetic resonance imaging (MRI), and tumor extent was delineated for surgical planning.

The planned resection margin was set 0.5 cm proximal and distal to the lesion to preserve bone stock; intraoperative microwave therapy was applied to further enlarge the effective ablation/resection zone. The intramedullary pin of the mortise‐tenon diaphyseal prosthesis was custom‐designed based on the measured length and diameter of the medullary cavity after osteotomy. For patients without pathological fractures, the risk of impending fracture was assessed using the Mirel's score [17].

### 2.4 Prosthetic Design

**FIGURE 1:**
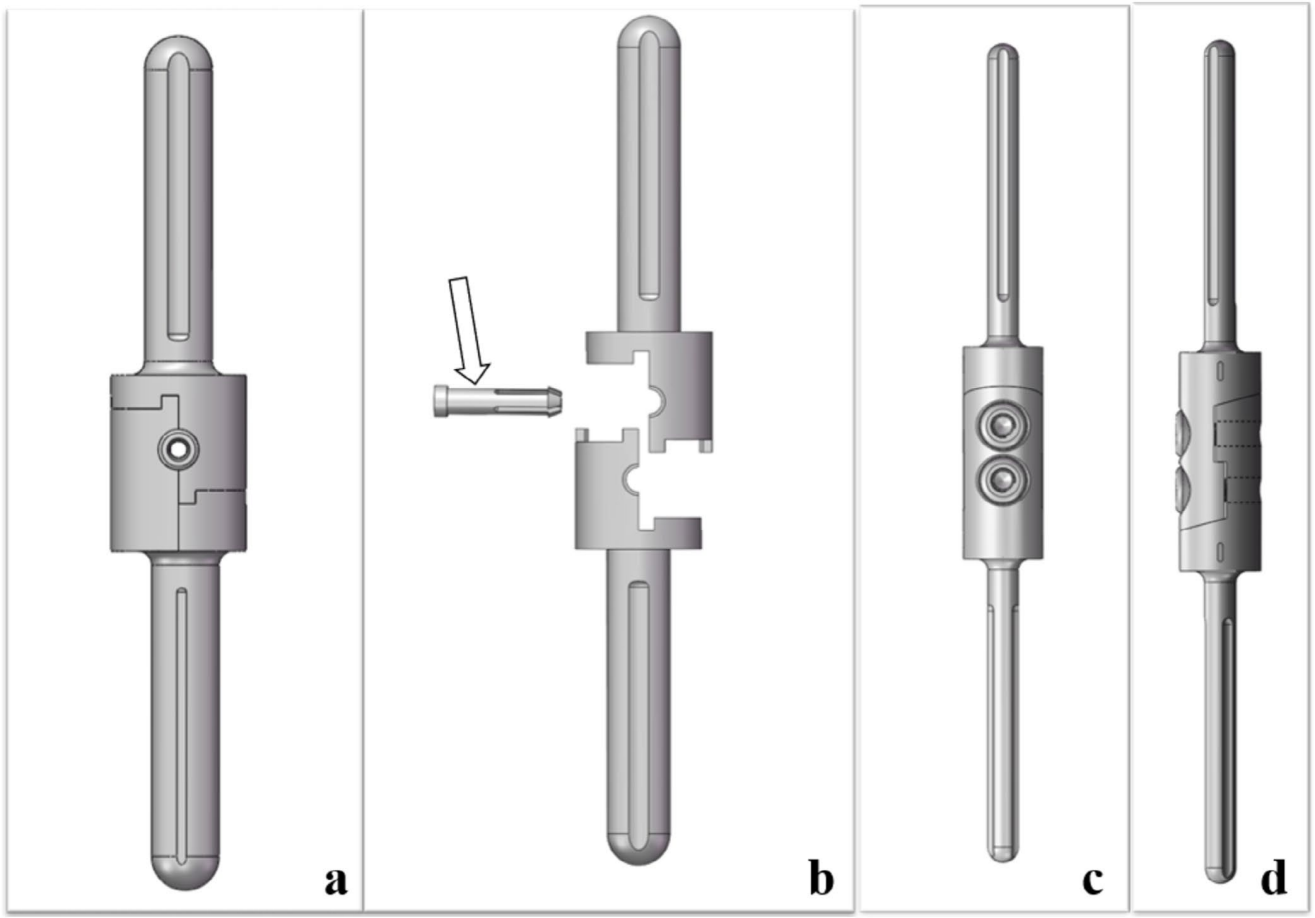
(a) Assembled and (b) disassembled components of the diaphyseal mortise‐tenon prosthesis, including a distal stem, a proximal stem, and a tenon. The tenon (arrow) includes a tenon cap at one end and a cylinder with four barbs at the other end. (c and d) The traditional modular diaphyseal prosthesis is fixed with two intermediate screws in the middle.

The diaphyseal mortise‐tenon prosthesis used in this study was designed and manufactured by Wego (Beijing, China). The design is covered by a Chinese patent application (No. CN201710751694.6). The implant was composed of proximal and distal components, which were connected through a mirror‐symmetrical interface. With reference to the traditional mortise–tenon joint, interlocking between the two components was achieved via precisely matched protrusions and grooves, and fixation was secured using a tenon consisting of a cap and a cylindrical body. A central circular opening in the tenon cap was aligned with the cylindrical body. The tenon cylinder was equipped with four evenly spaced feet, each terminated by a barb to enhance fixation. After tenon insertion, expansion of the barbed feet was achieved, by which engagement with the prosthesis was created and dislodgement was resisted (Figure 1).

For revision procedures, the tenon could be disengaged by removal of the cap locking screw using the manufacturer‐provided driver, followed by extraction of the tenon in the reverse direction with the dedicated instrument set.

### 2.5 Surgical Procedure

Patients were positioned supine, and a dorsolateral forearm approach was used. Incision length was determined according to the extent of tumor involvement identified on preoperative MRI. The skin, subcutaneous tissue, and deep fascia were incised sequentially. For ulnar diaphyseal lesions, exposure was achieved through dissection along the interval between the flexor carpi ulnaris and extensor carpi ulnaris; for radial diaphyseal lesions, exposure was obtained through the interval between the brachioradialis and extensor carpi radialis longus and brevis. By this approach, direct access to the diaphyseal tumor was obtained. Intraoperative microwave hyperthermia was applied as a local adjuvant to assist with margin control.

The resection length and osteotomy plane were determined according to the preoperative plan, and the periosteum was elevated at the planned osteotomy level. Periosteal elevators were placed on both sides to protect the surrounding soft tissues. The tumor‐bearing segment was excised en bloc and submitted for pathological examination.

**FIGURE 2:**
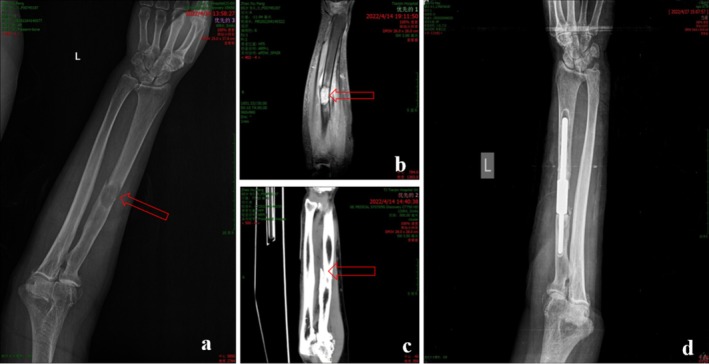
The diaphyseal mortise‐tenon prosthetic reconstruction for pathological fracture of the left radial diaphysis in patient 1. (a and b) Preoperative X‐ray and magnetic resonance images show metastatic lesion in the left radius (*arrows*). (c) Preoperative computed tomography scan shows osteolytic destruction, discontinuity of the bony cortex, an obvious fracture line, and angulation deformity in the left radial diaphysis (*arrow*). (d) Postoperative X‐ray image shows that the prosthesis is in good position. All images have been anonymized to protect patient privacy.

After gradual intramedullary canal preparation, including progressive reaming, irrigation, and hemostasis, bone cement was injected into the medullary cavity using a spinal PVP puncture needle. After alignment was confirmed, the proximal and distal prosthetic segments were connected and secured using the mortise–tenon mechanism, and the construct was held until cement polymerization and solidification were completed. The tourniquet was then released, meticulous hemostasis was performed, and a negative‐pressure drainage tube was placed. Finally, the incision was closed in layers (Figure 2).

### 2.6 Postoperative Treatment and Follow‐Up

The negative‐pressure drainage tube was removed when the daily output was < 50 mL for two consecutive days. Intravenous antibiotics were administered for 2–4 days postoperatively for infection prophylaxis, and functional rehabilitation exercises were initiated immediately after drain removal. Skin sutures were removed 12–14 days after surgery.

Adjuvant treatments were administered according to the primary tumor type: radiotherapy combined with chemotherapy was administered in one patient, chemotherapy alone was administered in one patient, endocrine therapy was administered in one patient, targeted therapy was administered in one patient, and targeted therapy combined with immunotherapy was administered in one patient. Outpatient follow‐up visits were scheduled every 3 months for the first year and every 6 months thereafter. At each visit, anteroposterior and lateral radiographs were obtained to monitor for complications and implant status, and color Doppler ultrasonography was used to assess periprosthetic soft tissues and screen for suspected local recurrence. Local recurrence was defined as a new or progressive lesion at or adjacent to the resection site on imaging; when findings were suspicious, additional CT and/or MRI was obtained as clinically indicated.

Pain was assessed using the VAS score [18], with higher scores indicating greater pain. Upper‐extremity function was evaluated using the MSTS functional score [19], with higher scores reflecting better upper extremity function (Table 2).

**TABLE 2:** Postoperative data of patients.

Patient	Follow‐up (months)	Adjuvant therapy	Complications	Outcome	VAS score	MSTS score
1	27	Radiotherapy and Chemotherapy	None	DOD	1	26
2	48	Endocrine therapy	None	AWD	0	27
3	19	Targeted therapy	None	DOD	0	25
4	35	Targeted therapy and Immunotherapy	None	DOD	1	27
5	72	Chemotherapy	None	AWD	0	26

Abbreviations: AWD, alive with disease; DOD, dead of disease; MSTS, Musculoskeletal Tumor Society; VAS, visual analogue scale.

### 2.7 Statistical Analysis

All statistical analyses were performed using SPSS software (version 27.0; IBM Corp., Armonk, NY, USA). Given the small sample size of this study (*n* = 5), the power of the normality test was considered insufficient; therefore, the Wilcoxon signed‐rank test was applied to compare preoperative and postoperative VAS pain scores as well as MSTS functional scores. A *p* value < 0.05 was regarded as statistically significant.

## 3 Results

En bloc resection of diaphyseal metastatic tumors was performed in all patients, and a surgical margin of 0.5 cm was applied. Follow‐up was achieved for 19–72 months (mean, 40.2 months). The mean osteotomy length was 3.6 cm (range, 3.0–4.0 cm). Three patients presented with pathological fractures preoperatively, while the remaining two had Mirel's scores of 10. At the final follow‐up, three patients had died of disease (DOD) and two were alive with disease (AWD).

**FIGURE 3:**
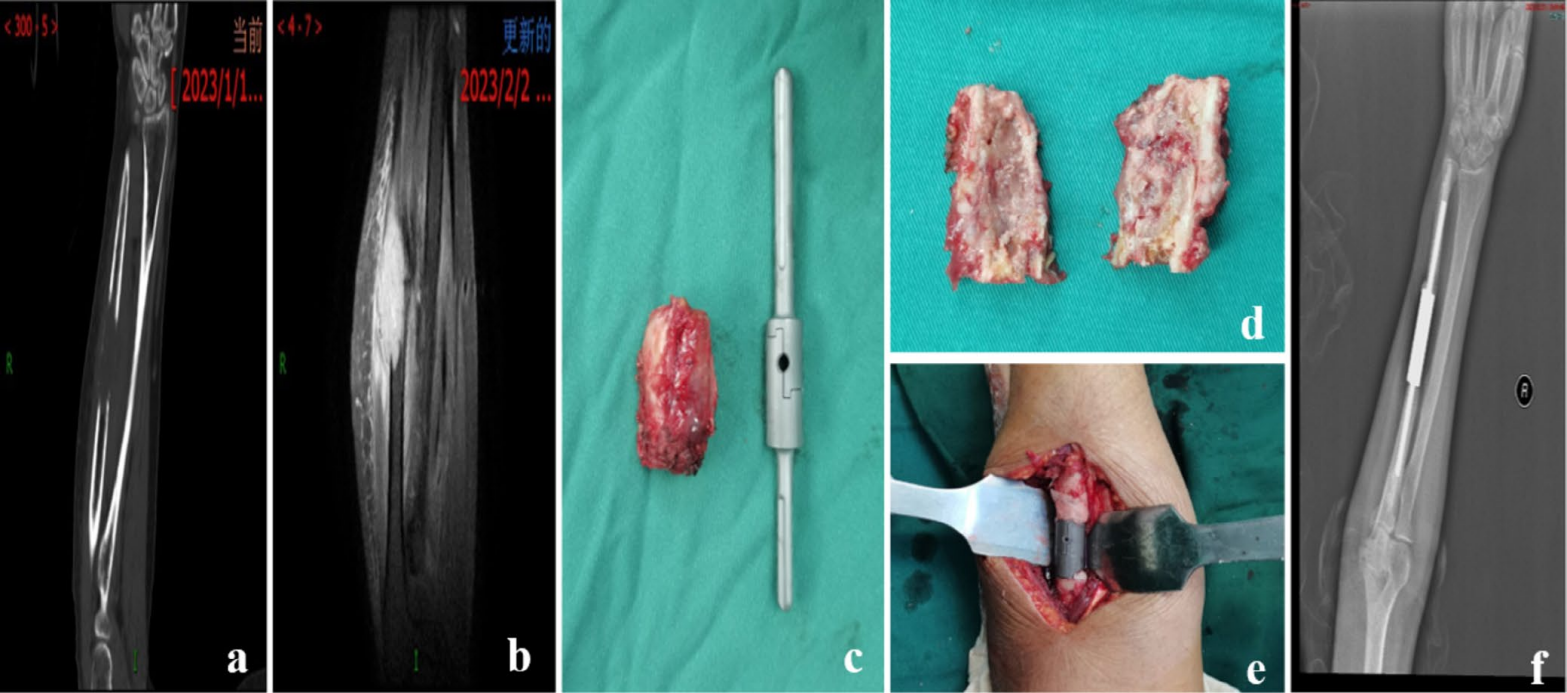
A diaphyseal mortise‐tenon prosthesis was implanted in patient 2 for a metastatic lesion of the right ulnar diaphysis. Preoperative (a) computed tomography scan and (b) MR image show metastatic lesion of the right ulnar diaphysis. (c and d) The resected tumor segment and the customized diaphyseal mortise‐tenon prosthesis are shown. (e) Intraoperative photograph shows implantation of a diaphyseal mortise‐tenon prosthesis. (f) Postoperative X‐ray image shows that the prosthesis is in good position.

**FIGURE 4:**
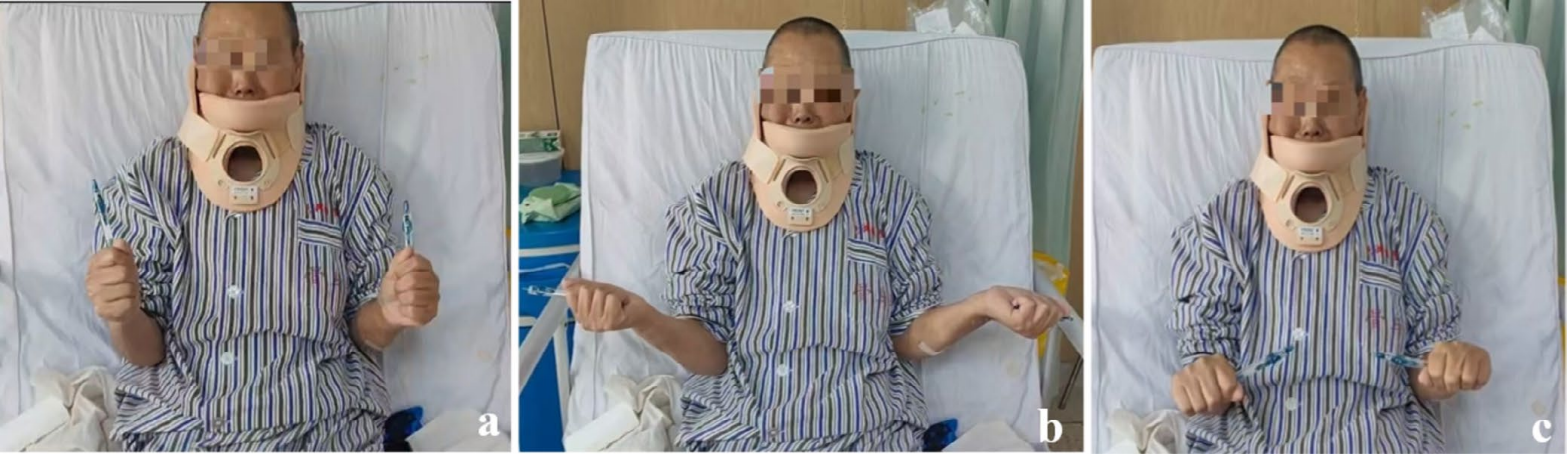
Patient 2 developed thoracic vertebral metastasis of breast cancer at 18 months postoperatively and underwent surgical treatment and there was no recurrence in the right ulna. The photos of forearm function showed satisfactory functional outcomes, including (a) neutral position, (b) supination, and (c) pronation.

No local recurrences, implant failures, or other postoperative complications were observed. Specifically, no wound complications or infections occurred, and no reoperation was required during follow‐up (Figure 3). Thoracic vertebral metastasis from breast cancer was detected in one patient (Patient 2) at 18 months postoperatively, and additional surgical treatment was performed; no recurrence was identified at the original ulnar site (Figure 4).

**FIGURE 5:**
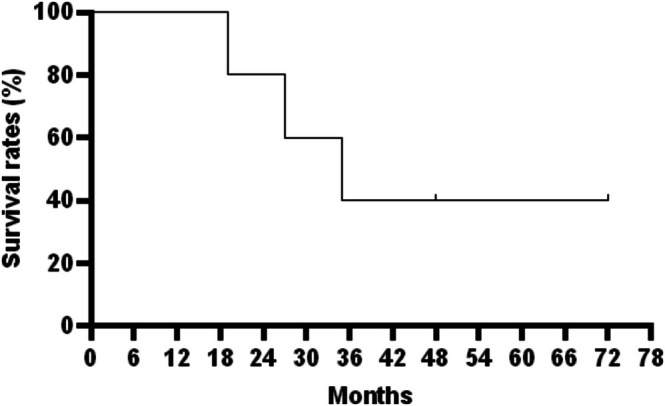
Survival time of patients with forearm diaphyseal bone metastases treated with en bloc resection and the mortise‐tenon diaphyseal prosthesis implantation. The 12‐month survival rate was 100%, and the 24‐month survival rate was 80%.

At the last follow‐up, the mean postoperative VAS score was 0.4 (range, 0–1) and the mean MSTS score was 26.2 (range, 25–27). Both scores demonstrated significant improvement compared with preoperative values (*p* = 0.034 and *p* = 0.039, respectively; Table 3). The 12‐month and 24‐month survival rates were 100% and 80%, respectively (Figure 5).

**TABLE 3:** Comparison of preoperative and postoperative pain and functional scores.

Item	Preoperative VAS score	Postoperative VAS score	Preoperative MSTS score	Postoperative MSTS score
No. 1	9	1	20	26
No. 2	8	0	19	27
No. 3	8	0	18	25
No. 4	7	1	19	26
No. 5	8	0	18	27
*p* value	0.034		0.039	

Abbreviations: MSTS, Musculoskeletal Tumor Society; VAS, visual analogue scale.

## 4 Discussion

### 4.1 Main Findings

This single‐center case series evaluated intercalary reconstruction using a mortise–tenon diaphyseal prosthesis after en bloc resection for forearm diaphyseal metastatic defects. Ultra‐short osteotomy was achieved (mean, 3.6 cm), postoperative pain and function improved (VAS and MSTS), and no local recurrence or implant‐related failure was observed during follow‐up (Table 4). These early results support the feasibility of this reconstruction in carefully selected patients.

**TABLE 4:** Comparison of current study results with those of other studies involving surgical treatment for forearm bone metastatic lesions.

Author	Publish date	Case (*N*)	Age (year)	Primary tumor	Surgical method	Osteotomy length (cm)	Complications	Follo w‐up (months)	Survival time	MSTS score
Ratasvuori [12]	2013	14	N/A	Variety of cancers	Plate and nail Prothesis Other	N/A	N/A	N/A	N/A	N/A
Huang [20]	2014	11	N/A	Variety of cancers	Curettage + bone cement filling + plate/intramedullary nail internal fixation Segment resection + artificial elbow joint replacement Segmental resection	N/A	None	N/A	N/A	21.36
Tang [21]	2018	21	60.9	Variety of cancers	Artificial elbow joint replacement Curettage + bone cement filling + plate internal fixation	N/A	Poor healing of superficial wounds (2)	8 (MT)	7 (MT)	22.5
Zheng [22]	2019	1	> 80	Undifferentiated sarcoma	Wide resection with modular prosthesis reconstruction	6.0	None	38	N/A	28
Hashimoto [23]	2024	10	69.3	Variety of cancers	IMN Plating	N/A	None	8	26.3	25.6
Sebghati [24]	2025	28	71 (MA)	Variety of cancers	Plate osteosynthesis Segmental resection Curettage Amputation Other (pins)	N/A	Tumor growth (3) Collapse (2) Local infection (1)	11 (MT)	24	N/A
Our study	2025	5	68.4	Variety of cancers	En‐bloc resection with mortise‐tenon diaphyseal prosthesis reconstruction	3.6	0	40.2	27	26.2

Abbreviations: IMN, intramedullary nail; MA, median age; MSTS, Musculoskeletal Tumor Society; MT, median time; N/A, not applicable.

### 4.2 En Bloc Resection Versus Intralesional Procedures in Forearm Metastases

In clinical practice, forearm metastases have been managed using plate or intramedullary nail fixation and intralesional procedures such as curettage with cement filling. However, when fixation or intralesional surgery is performed, tumor violation is generally unavoidable, and incomplete clearance may be introduced, potentially increasing the risk of local progression and subsequent mechanical or wound‐related complications. Sebghati et al. [24] reported complications in five of 28 patients treated with plate fixation or curettage, including two cases of collapse (one with concurrent infection) and three cases of tumor growth. Similarly, Tang [21] documented poor wound healing in patients who underwent curettage. By contrast, en bloc resection provides better local control and has been associated with improved survival in patients with solitary skeletal metastases [25, 26]. In the present series, a low rate of postoperative complications was observed after en bloc resection followed by prosthetic reconstruction, although direct comparison with other approaches could not be performed because a control group was not available.

### 4.3 Comparison With Biological and Other Reconstructive Options

After en bloc resection of bone tumors, biological reconstructions including allografts [27, 28] and vascularized fibula grafts [29, 30] have been commonly employed. For allografts, infection, fracture, and nonunion have been frequently reported, and additional procedures may be required to address these complications. Reported rates have ranged from 12% to 14% [31, 32] for infection 9% to 19% [33, 34], and 17% to 50% for nonunion [31, 32, 35]. Similarly, vascularized fibular grafting has been associated with delayed union or nonunion, infection, fatigue fracture of the graft, and donor‐site morbidity [30, 36]. In addition, the initiation of rehabilitation is often delayed because a prolonged bone‐healing phase is required. Alternative techniques have been described in selected scenarios, such as radial neck‐to‐humerus trochlea transposition [13], although applicability has been limited to proximal ulna reconstruction and long‐term outcomes remain unclear. Minimally invasive fixation devices have also been explore [37], yet their oncologic and mechanical efficacy compared to conventional methods has not been firmly established. Amputation results in complete functional loss and is generally reserved for highly selected circumstances.

### 4.4 Rationale for Endoprosthetic Reconstruction and Implications of Ultra‐Short Osteotomy

In this context, endoprosthetic reconstruction has been increasingly adopted because immediate structural stability can be provided, early mobilization can be facilitated, and the prolonged biological healing phase can be avoided [38, 39]. Evidence from diaphyseal humeral reconstructions has suggested that endoprosthetic replacement may provide more reliable resistance to extrusion, bending, and torsional loading than internal fixation or biological reconstruction [14, 15, 40]. Nevertheless, conventional diaphyseal prostheses often require long osteotomy lengths (frequently > 6 cm for the ulna, > 8 cm for thehumerus [22, 38]), because overlapping fixation segments and intermediate screws require substantial bone stock. It has been suggested that the remaining proximal and distal bone segments after osteotomy should not be shorter than 5 cm to allow cement fixation [41], and a minimum intramedullary fixation length of 5 cm has also been recommended, as early loosening may occur when fixation length is < 5 cm [42]. These requirements may be difficult to satisfy in the radius and ulna, where lesions are often limited in length and available diaphyseal segments are short.

To mitigate this limitation, a compact interlocking mechanism was incorporated into the present mortise–tenon design, by which segmental connection can be achieved without bulky overlapping stems. By this configuration, the osteotomy length was reduced to approximately 3–4 cm, bone stock was preserved, and intraoperative assembly was simplified because repeated adjustments and intermediate screw fixation were not required. In addition, the four‐barbed tenon feet were designed to enhance fixation and reduce the risk of dislodgement, which is a potential concern in modular constructs. While these design features are conceptually advantageous, their mechanical superiority relative to conventional systems should be further validated.

### 4.5 Limitations and Prospect

Several limitations of this study should be acknowledged. First, the sample size was small (*n* = 5), reflecting the rarity of forearm diaphyseal metastases, and the absence of a control group precluded direct comparison with other reconstruction methods. Second, although follow‐up extended to 72 months, the duration may still be insufficient to fully characterize long‐term prosthetic survival and late complications. Accordingly, multicenter studies with larger cohorts and comparative designs are warranted to further evaluate durability, oncologic safety, and functional outcomes of mortise–tenon diaphyseal prosthetic reconstruction, and to explore whether this ultra‐short osteotomy strategy can be applied to other anatomical sites requiring short‐segment diaphyseal replacement.

## 5 Conclusion

In summary, mortise‐tenon diaphyseal endoprosthetic reconstruction with an ultra‐short osteotomy may be a feasible option for selected patients with forearm diaphyseal metastatic defects after en bloc resection. Further studies are needed to confirm long‐term durability and to clarify its comparative effectiveness against other reconstruction strategies.

## Author Contributions

**Yunxiu Chen** and **Tongfu Wang** contributed equally to this work. **Chengke Li** and **Jingyu Zhang** conceived and designed the study. **Miaomiao Gao** and **Xiaoyi Wang** performed the data analysis. **Yunxiu Chen** and **Tongfu Wang** drafted the initial manuscript. All authors critically revised the manuscript for important intellectual content and approved the final version.

## Funding

This work was supported by Tianjin Hospital Science and Technology Fund (TJYY2401) and Science and Technology Project of Tianjin Health Commission (TJWJ2024MS027).

## Ethics Statement

The study was approved by the Institutional Review Board of Tianjin Hospital (Approval No. 2024 Medical Ethics Bureau Pre‐020), and informed consent was obtained from all participants.

## Conflicts of Interest

The authors declare no conflicts of interest.

## Data Availability

The data that support the findings of this study are available on request from the corresponding author. The data are not publicly available due to privacy or ethical restrictions.
